# Geographic Information System and tools of spatial analysis in a pneumococcal vaccine trial

**DOI:** 10.1186/1756-0500-5-51

**Published:** 2012-01-20

**Authors:** Antti Tanskanen, Leilani T Nillos, Antti Lehtinen, Hanna Nohynek, Diozele Hazel M Sanvictores, Eric AF Simões, Veronica L Tallo, Marilla G Lucero

**Affiliations:** 1 National Institute for Health and Welfare, Helsinki, Finland; 2 Research Institute for Tropical Medicine, Muntinlupa City, Philippines; 3 School of Economics, Aalto University, Helsinki, Finland; 4 University of Colorado, Denver, USA

## Abstract

**Background:**

The goal of this Geographic Information System (GIS) study was to obtain accurate information on the locations of study subjects, road network and services for research purposes so that the clinical outcomes of interest (e.g., vaccine efficacy, burden of disease, nasopharyngeal colonization and its reduction) could be linked and analyzed at a distance from health centers, hospitals, doctors and other important services. The information on locations can be used to investigate more accurate crowdedness, herd immunity and/or transmission patterns.

**Method:**

A randomized, placebo-controlled, double-blind trial of an 11-valent pneumococcal conjugate vaccine (11PCV) was conducted in Bohol Province in central Philippines, from July 2000 to December 2004. We collected the information on the geographic location of the households (N = 13,208) of study subjects. We also collected a total of 1982 locations of health and other services in the six municipalities and a comprehensive GIS data over the road network in the area.

**Results:**

We calculated the numbers of other study subjects (vaccine and placebo recipients, respectively) within the neighborhood of each study subject. We calculated distances to different services and identified the subjects sharing the same services (calculated by distance). This article shows how to collect a complete GIS data set for human to human transmitted vaccine study in developing country settings in an efficient and economical way.

**Conclusions:**

The collection of geographic locations in intervention trials should become a routine task. The results of public health research may highly depend on spatial relationships among the study subjects and between the study subjects and the environment, both natural and infrastructural.

**Trial registration number:**

ISRCTN: ISRCTN62323832

## Background

Many vaccine trials have been based on individual randomization with an assumption that factors affecting vaccine efficacy is balanced. More precisely, this assumption presumes a random distribution of the environment and population as well as an equal force of infection among vaccinated and control subjects. The spatial distributions of vaccine and control recipients are assumed to be random. Clustering due to varying vaccine coverage in the immediate neighborhood is usually not considered. To measure the spatio-temporal effects of vaccination the time-dependent process of vaccination needs to be related to the location of the study subjects. Emch et al. [1] showed that vaccine efficacy may show considerable variation locally. For example, variation in the density of population may influence transmission rates and thus the measures of vaccine efficacy [2]. In addition, Moïsi et al. [3] have shown that the vaccination status of the population not attending to the trial should also be considered. In reality, few vaccine trials are measuring pure direct vaccine efficacy. More often vaccine efficacy is impacted by hard to control for factors which may have spatio-temporal dimensions. These factors include for example herd immunity, local epidemics and population density. The main outcome of trials in such circumstances is better defined as vaccine effectiveness.

The endpoints of interest measured in vaccine trials are often obtained via passive surveillance systems that are recorded during outpatient or hospital visits due to illness. Access to care varies locally and this can affect the rate and/or severity of symptoms recorded in the study subjects. One measure of access is the travel effort [4,5] or the geographic distance to health services [6]. Results may also be affected by the local socio-economic conditions, women's social status [7] or patterns of health behavior. Some of these factors may depend on the spatial factors such as crowdedness. Thus, the human-to-human transmission potential of pathogens may be locally higher close to the services, and this needs to be taken into account when interpreting results of trials and other intervention studies.

To enhance the understanding and appreciation on how to obtain and utilize spatial data in a trial context, we describe the Acute Respiratory Infection Vaccine (ARIVAC) consortium experience. Geographic Information System (GIS) data were collected from the area where a phase III trial, i.e. an individual-randomized, placebo-controlled, double-blind efficacy trial of an investigational 11-valent pneumococcal conjugate vaccine (11PCV; ISRCTN 62323832) was carried out. The trial was conducted in 6 municipalities (Tagbilaran City, Dauis, Panglao, Baclayon, Cortes, and Balilihan) in Bohol Province of the central Philippines, from July 2000 to December 2004 [8], in children aged from 6 weeks to 6 months. The primary objective of the core trial was to determine the vaccine efficacy (VE) against the first episode of radiologically confirmed pneumonia in the first 2 years of life. Infants and children under 2 years of age, who lived in the same study areas but were not enrolled in the core trial, were part of an epidemiological study. Here we describe the process of creating reliable GIS data in a setting of a resource poor country without existing spatial information. This data will be used to answer various a posteriori research questions that may arise in the vaccine trials.

## Methods

GIS data were collected to obtain information on the location of the households of study subjects in the core trial (both 11PCV and placebo recipients) and in the epidemiological study. The 6 municipalities were divided into 48 smaller administrative units called barangays. Information on the locations of barangay (village or city district) health stations, health centers, hospitals and other landmarks as well as the road network in the six municipalities was obtained. The data were collected from November 2008 to June 2009. We used Arc/Info Workstation (ESRI Inc.) for the GIS calculations, Manifold 8 http://www.manifold.net for the spatial illustrations and SPSS 17 (SPSS Inc.) and SAS 9.2 (SAS Institute Inc.) for the statistical calculations. The GIS study was part of the Herd immunity sub-study protocol and along with the core trial was approved by the ethics review board of the Research Institute for Tropical Medicine (RITM), Philippines and of National Institute for Health and Welfare (THL), Finland.

### Field data collection

The location of the birth addresses of 12,194 children enrolled in the efficacy trial and the 577 children enrolled in the epidemiological study were collected with a hand-held Global Positioning System (GPS) unit Magellan Triton 500^R^, marking a waypoint at each location. If the child moved before the age of 2 years, an additional waypoint was collected. Six field workers trained to use the GPS units and the Vantagepoint software collected the waypoints. A pre-completed collection form was prepared from the trial database to guide the field worker in locating households where the children lived at birth and at 2 years of age. The form contained the demographic information of the child, the maiden name of the mother, the address of the household at the time of enrolment, whether the child completed or was withdrawn from the trial, and any amendments to the information of the child after enrolment. The form was also used to input the GIS data (latitude and longitude), whether the waypoints were the exact locations at birth and at 2 years of age, to complete the coordinates saved in the GPS hand held unit and to have a paper backup. The location of the household was confirmed by the mother or family members and by neighbors, if the family was not available or had moved. If it was not possible to measure the exact location accurately, the place was flagged as an estimate in the collection form. If the child moved out from the study area and transferred to a different location, the field worker attempted to determine the new location. The field worker wrote the household waypoints and relevant information on the collection form for documentation.

Besides the locations of the households of the study children, relevant landmarks such as hospitals, health stations, health care providers, schools, offices and businesses where people spend their time outside their home were also collected. The road networks were collected with no classification to major or minor ones.

During the first visits to the barangays, the field worker introduced themselves to the heads of the barangay and informed them of the activities to be done. They also gathered some general information about the area, such as the number of barangay health workers (BHWs), number of establishments, and the availability of private healthcare providers. The field worker also solicited the help of the BHWs who were familiar with the area, location of the households and information regarding the families. Some of them were also involved in recruitment and enrolment of the children to the original trial.

Depending on the area, the field worker either collected the information alone, in pairs with a colleague or accompanied by the BHW. Field work was done municipality by municipality. Approximately 50% of the children enrolled to the core trial resided in the capital of the province, Tagbilaran City.

The waypoints for certain landmarks, such as schools and health services, were collected simultaneously with the household data. Each landmark was assigned a specific type. There was no decent roadmap available of the study area. To get the GPS tracks of the roads, the field worker either rode a motorcycle or walked through the roads. The GPS tracks were saved as trails when the tracking ended or if the GPS lost satellite connection.

After each working day, the field worker transferred the GIS data from the GPS to a dedicated computer in the project office. Every Monday, data accompanied with a weekly report of the field activities were transmitted to the Research Institute for Tropical Medicine (RITM), Manila. The weekly reports included status of completion, problems encountered and updates on the waypoints previously collected.

We used a given naming practice for the waypoints, landmarks and trails. The waypoint identification (ID) from the GPS unit was a running number from 001 after every reset of GPS, which was done daily. Since this kind of numbering was difficult to identify, we replaced it by the child identification number assigned to the child at the time of trial enrolment. If a different waypoint was collected for the child at the age of 2 years, the identification number was appended with the letter 'A'. If the child moved to a different location before reaching the age of 2 years, the waypoint ID was appended with the letter 'B'. A unique codename was assigned to each unique landmark. The naming practice was based on the landmark types (Table 1) followed by the municipality and barangay identification numbers, a three digit chronological number and the field worker ID of the person who collected the landmark points. The trail names started with letter 'T' that was followed by the municipality and barangay identification numbers, a three digit chronological number and the field worker ID of the person who collected the trail waypoints.

**Table 1:** Distribution of the various landmark types in the study area

Landmark	Number (%) N = 1,982
**Administrative**	**340 (17.15)**
Barangay hall	109 (5.50)
Fire station	1 (0.05)
Government/Private office	13 (0.66)
Municipal office	4 (0.20)
NAPOCOR (National Power Corporation)	1 (0.05)
Prison	2 (0.10)
Purok (Zone)	210 (10.60)

**Commercial**	**319 (16.09)**
Commercial building	34 (1.72)
Daily supply	115 (5.80)
Hardware/construction supply	30 (1.51)
Health related	9 (0.45)
Hotel/resort/Lodge/Inn	36 (1.82)
Special	37 (1.87)
Transportation distributor	58 (2.93)

**Education**	**226 (11.40)**
Convent/Seminary/Monastery	5 (0.25)
Daycare center	36 (1.82)
Orphanage	1 (0.05)
Reservoir	8 (0.40)
School	72 (3.63)
Sports field	104 (5.25)

**Health service**	**775 (39.10)**
Barangay health station	48 (2.42)
Doctor's house	1 (0.05)
Hospital	8 (0.40)
Pharmacy	1 (0.05)
Private clinic	10 (0.50)
Sari-sari store (Variety store)	639 (32.24)
Water refilling station	13 (0.66)
Water well	55 (2.77)

**Religion**	**205 (10.34)**
Cemetery	7 (0.35)
Church/Chapel/Worship	198 (9.99)

**Transport**	**117 (5.90)**
Bridge	10 (0.50)
Harbor	1 (0.05)
Plaza	2 (0.10)
Terminal	4 (0.20)
Waiting shed	100 (5.05)

The study also collected a barangay profile to obtain information about the overall economic level, social welfare level, population and services available in the barangay. The respondents were the barangay captain, barangay councilor or BHW.

In addition to the 6 fieldworkers, the study involved one GIS officer in Bohol, one GIS officer in RITM, Manila and one researcher in National Institute for Health and Welfare (THL), Finland. Part of the processing of GIS data was performed by a GIS expert in School of Economics, Aalto University, Finland. Totally this work needed 65 person-months to be completed.

### Data validation and monitoring

The GIS officer in Bohol made all corrections in the data and she sent the corrected data to RITM. The GIS officer in RITM merged the incoming GIS data so that three data files were produced for each barangay: one for the household waypoints, one for the landmarks and one for the roads. For the merging, GPSbabel http://www.gpsbabel.org/ and EasyGPS http://www.easygps.com software were used. The GIS officer also transformed the GIS data into a SAS (SAS Institute Inc.) dataset to check for possible queries and problems in the waypoints. In case of such problems, she submitted the data back to the field worker for correction and verification. Some queries included duplicate waypoints and incorrect waypoint names. The correction process led to the re-locating of the households and landmarks, re-tracking of the roads and modification or deletion of the collected waypoints. The merged and corrected data were stored in GPS exchange (GPX) formats and were sent from RITM to THL.

A random sample (67 subjects) was selected for a quality control check to validate the household waypoints collected previously by the fieldworkers from the 5 municipalities (Dauis, Panglao, Baclayon, Cortes and Balilihan). This random sample was taken from all collected waypoints of each fieldworker and recollected. The RITM GIS officer visited the study area to obtain feedback from the field workers and to verify queries from previously submitted waypoints.

### Spatial data

The raw version of the GIS data was not suitable for analysis as such and required major processing. This process is described below.

#### Road network

We used the Arc/Info Workstation for preparation of the geographic data and calculation of the distance metrics. The GPS-based data, recorded as geographic coordinates with decimal degrees, were imported to the Arc/Info and projected to UTM zone 51 projection. The most demanding task in post-processing of the data was required for cleaning of the road network tracks. The data contained multiple overlapping tracks as well as errors due to poor signal, such as gaps or road intersections, where the tracks did not actually cross.

First, a large number of overlapping tracks were deleted from the network. We then artificially increased the spatial resolution of the tracks by introducing additional points into the lines for every 10 m. To get a preliminary version with most intersections correctly modeled, we cleaned the road layer with a fuzzy tolerance of 5 m, which effectively collapsed all points within the tolerance into a single point. Most gaps were then closed by testing, whether there was any intersecting line within 20 m from the end points of any line, which was not connected to any other line (a dead end).

Many of the roads in the study area were recorded in both directions. The distance between separate lanes in addition to the GPS accuracy, resulted in two parallel lines, which in some cases were further apart than the 5 m tolerance. We converted the road layer to a raster dataset with a cell size of 1 m. An iterative loop of expanding and thinning the road areas was applied to collapse the separate parallel lines to a single centerline representing roughly the weighted average of the original GPS tracks. This raster dataset was re-vectorized and the intersection node points were snapped to the intersection points of the pre-rasterization layer.

A total of 17 gaps or other errors were too large to fit within the tolerances of the automated cleaning process and needed to be edited manually. To reduce the remaining measurement noise, we generalized the road network using the shape preserving Douglas-Peucker algorithm [9]. Finally, the topology of the road network was built and checked. The result was a fully connected GIS dataset that could be used for route optimization and calculation of the true network distances. The final results were manually checked and corrected by using available satellite images and maps.

#### Study subjects and network analysis

The GIS point layers were created from the GPS data, containing the locations of homes of the study subjects and the landmarks classified in 60 classes (Table 1). Each point in the data includes the latitude and longitude coordinates as well as the ID and type of the point. We calculated the closest location along the road line for every object of the point layers, effectively connecting the points to the road network.

To overcome problems in the poor altitude resolution of the GPS data, we selected to use the ASTER GDEM^1 ^digital elevation model to discover the elevation of the homes of the study subjects. We overlaid the point database of the study subjects on the elevation model within the Arc/Info GRID-module and added the elevation as an attribute to the point database.

For all study subjects, we identified the closest landmark of importance. This was calculated on multiple levels of the health facilities and public gathering places with the Arc/Info Allocate command, which utilized the road layer for true network distances. The study area consists of two islands connected by two bridges creating a geographic setting, where the use of the Euclidean measurements for the analysis would be justified only over short distances.

#### Spatial relationships

The immediate surroundings of every study subject were analyzed from two separate points of view by utilizing Euclidean distances. First, we used the buffer radii of 10, 100, 250, 500, 1000 and 2000 m within which we calculated the numbers of the 11PCV and placebo recipients during the whole study. Additionally, we calculated the numbers of 11PCV and placebo recipients that had been born before the subject in question was born. We also recorded the number of landmark objects as a proxy for the level of services available. For the purpose of normalization, each buffer zone was intersected by the shore line and the remaining land area was recorded. Secondly, for each study subject we identified and calculated the distance to the closest 11PCV and placebo recipient without any cut-off distance.

## Results

The original study population consisted of 12,771 children living in 10,607 households (Table 2). There were 8565 (80.7%) households with one child belonging to study population, 1923 (18.1%) households with 2 children, 116 (1.1%) households with 3 children and 3 (0.0%) households with 4 children, respectively.

**Table 2:** Characteristics of the six municipalities by land area, population (year 2000) and number of study children enrolled in the study.

Characteristics	Tagbilaran	Dauis	Panglao	Baclayon	Cortes	Balilihan	Total
Land area (in km^2^)	30.61	42.88	55.37	34.02	43.77	150.22	356.87
Total population	77, 700	26, 415	21,337	14,996	12,702	16,837	169,987
PNF13 and EPI children enrolled	6,102	2,259	1,564	846	926	1,074	12,771
**Waypoints collected**							
**Households**							
At birth	5,784	2,234	1,551	835	919	1,060	12,383
Different at 2 years old	437	101	45	46	77	93	799
Transient	7	6	1	1	6	5	26
**Landmarks**	624	607	260	102	191	198	1,982

For each municipality there are the numbers of household locations, where the study children were born, and the number of household locations where the study children were living at the age of 2 years. Transient children (not permanently living in area) were separately counted

**Figure 1:**
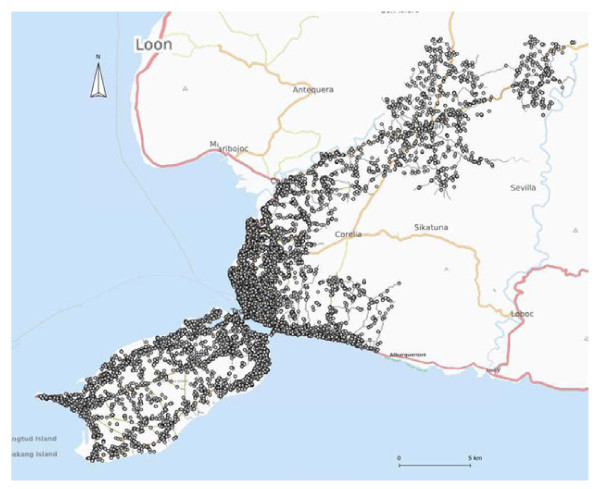
**The locations of households at birth (12,383) in the study area Background map: www.openstreetmap.org**.

A total of 13,208 locations (waypoints) were collected with 12,383 at birth (93.8%), 799 (6.0%) at the age of 2 years and 26 (0.2%) transients or those who moved away before reaching the age of 2 years (Figure 1). We did not find the place of residence at birth for 383 children and at the age of 2 years for 992 children. Reasons for not collecting these locations were as follows: 1) not found at birth; 2) lived elsewhere in Bohol at birth; 3) lived outside Bohol at birth; 4) not found at 2 years of age; 5) lived elsewhere in Bohol at 2 years of age; 6) lived outside Bohol at 2 years of age; and 7) died before 2 years of age.

**Figure 2:**
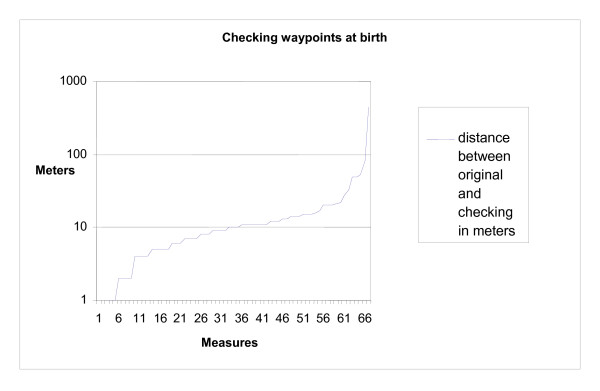
**Random checking of 67 household waypoints at birth**.

Out of the 67 subjects selected for the quality check, in 57 (85.1%) cases the re-located and the original locations were in good agreement. In 7 (10.4%) cases, same locations were recorded at birth and at the age of 2 years in original collection while upon random checking different waypoints were collected at birth and at the age of 2 years. There were 3 occasions where different waypoints at birth and at the age of 2 years were originally collected, but according to checking child had the same place of living at birth and at the age of 2 years. One location recollected at birth was 430 m from the original one and the remaining 66 less than 100 m from the original (Figure 2).

**Figure 3:**
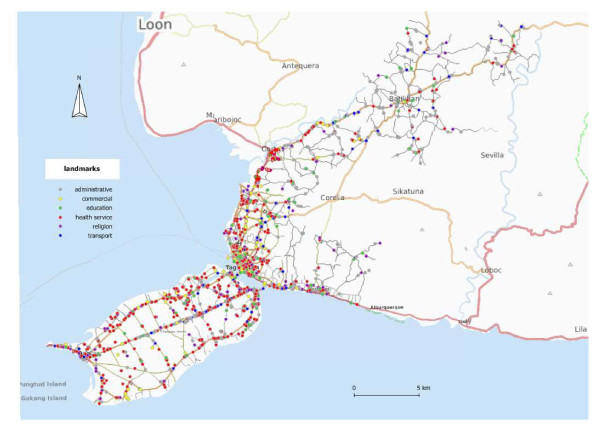
**Distribution of the landmarks and road network in the study area Background map: www.openstreetmap.org**.

We also collected 1982 landmarks. The landmarks were categorized into 60 types (Table 1). These can be grouped to six main types, namely the health services (775 (39.10%)), the educational (226 (11.40%)), the commercial (319 (16.09%)), the religion-related (205 (10.34%)), the transport (117 (5.90%)) and the administrative (340 (17.15%)) landmarks (Table 1 and Figure 3). The sari-sari or variety stores (belonging to the commercial landmarks) are the main source of antibiotics that are available without prescription.

There were 858 tracks in the original GPS data with 129,000 coordinate points. The total length of the original tracks was 1411 km. The length of processed final road network was 640 km.

The road network-based distance from the households of the study subjects to different services were grouped by the type of service (health services) or by the user of service (mother, older sibling, study subject). The median distance to the nearest sari-sari store was 212 m (range 0-6742 m, N = 5927) for the 11PCV recipients and 218 m (range 0-6811 m, N = 5905) for the placebo recipients. The median distance to nearest school (for possible older sibling) was 835 m (range 0-7284, N = 5927) for the 11PCV recipients and 813 m (range 0-6476 m, N = 5905) for the placebo recipients. All distances were positively skewed. The dense population in Tagbilaran City and its immediate surroundings with many services available produced many short distances, while fewer people living in remote areas had mostly long distances to many of these services.

For each child in the core trial, we determined the numbers of 11PCV and placebo recipients in a sequence of circular buffers with different radii around the household location at birth. Within a buffer of 500 m around the location of a study participant's household at birth, the numbers of 11PCV (N = 5927) and placebo (N = 5905) recipients were 0-328 (median 41) and 0-326 (median 41), respectively.

## Discussion

The ARIVAC trial was conducted from 2000 to 2004 and the geographic locations of the households of the participants in the core study were collected from 2008 to 2009. Although there was a 4 years gap, the collection of geographic locations was successful. This was mainly due to the good bookkeeping of BHS, good local knowledge of the BHS staff and the efficient communication of the fieldworkers in the neighborhood of the household of the presumed study child. After 4 years, 99% of families were still living in the same location as during the ARIVAC trial. The impact of new or changed services is unknown, but at least health services had not had essential changes during a 4 years gap between the completion of the ARIVAC trial and the collection of geographic data. Neither was any major change in the road network known to have occurred. The results showed that the data on geographic locations collected afterwards is reliable and fairly comprehensive.

The landmarks and the road network were easy to collect, while the collection of the household waypoints by the fieldworkers was more laborious. There were no decent maps available prior to data collection and no data could be digitized from the maps [10]. To make these new data available for the local community, all landmarks and the processed road network were donated to the OpenStreetMap project (see http://www.openstreetmap.org/?lat=9.6749&lon=123.9253&zoom=12&layers=M and http://wiki.openstreetmap.org/wiki/Talk:WikiProject_Philippines/Data_import_Arivac_Bohol). This made it possible to obtain a good roadmap of the area for free public use.

The GIS methods make possible the informative visualization of objects or events, such as clustering of diseases, variations in local vaccine coverage or population density. These visualizations might be valuable starting points for various preliminary analyses and useful ways to present the results of spatial analyses [11,12]. Maps are often much more understandable for laymen than large tables and thus more useful for policy-making [13].

Our approach makes it possible to measure the spatial characteristics on an individual level. It also makes possible to calculate the numbers of potential transmitters of the bacteria separately in the neighborhood of each study subject. In addition, connections of the households to relevant services (such as schools and shops) are possible with a distance metric. Thus, we can for instance estimate which families share the same services. By adding times of different events to these data, it is possible to analyze spatio-temporal processes with accurate data.

The spatial data were collected 5 years after original study. Services and road network have some expanded from the time of original study. Not all small roads were collected and many paths are missing. In spite of these limitations we believe that this data are fairly accurate and comprehensive and one of the largest collected in this kind of study.

## Conclusions

The collection of geographic locations in intervention trials could help to analyze and better understand spatial factors. The results of public health research may highly depend on spatial relationships among the study subjects and between the study subjects and the environment, both natural and infrastructural [1,14].

## Endnotes

^1^ASTER GDEM digital elevation model is the property of Japan's Ministry of Economy, Trade and industry (METI) and NASA

## Abbreviations

ARIVAC: Acute Respiratory Infection Vaccine Consortium; BHS: Barangay health station; BHW: Barangay health worker; EPI: Expanded program on immunization; GIS: Geographic Information System; GPS: Global Positioning System; GPX: GPS exchange format; ID: Identification; PCV: Pneumococcal conjugate vaccine; PNF13: 11PCV code; RITM: Research Institute for Tropical Medicine (Manila: Philippines); THL: National Institute for Health and Welfare (Helsinki: Finland); VE: Vaccine efficacy.

## Competing interests

The authors declare that they have no competing interests.

## Authors' contributions

AT participated in the study design, processed spatial data and drafted the manuscript. LTN and DHMS participated in the study design, supervised the data collection and assisted in drafting the manuscript. AL processed the spatial data. HN coordinated the intervention trial, participated in the study design, reviewed and provided comments to the manuscript. EAFS, VLT and MGL participated in the study design, reviewed and provided comments to the manuscript. All authors read and approved the final version of the manuscript for publication.
